# Practical Notes and Formulæ

**Published:** 1879-04-20

**Authors:** 


### PRACTICAL NOTES AND FORMULÆ.

**Habitual Constipation.**—Habitual constipation is a very common, and often a very difficult condition to overcome. Almost every journal contains formula and suggestions from the experience of practitioners promising much, but as a rule resulting in disappointment. We have for the last twelve months been using, with great satisfaction, minute doses of podophyllin, as contained in the *parvules* of Wm. R. Warner & Co., of Philadelphia. The parvules whieh we use contain only one-twentieth of a grain each, and we give only one pill regularly three ttmes a day. The podophyllin in powder does not seem to be so efficient. Why this is so, we cannot tell; perhaps it is the trituration, or something in the method of preparation; or it may be the purity of the article used by this staunch and reliable House, whose fame in the preparation of sugar-coated pills is world-wide. The effect of these parvules, given as above, is simply to re-establish and regulate the peristaltic action, producing one easy, slightly softened and apparently natural dejection daily. It also imparts tone to the liver, invigorates the digestive function, relieves hemorrhoids if they exist, and improves the general health.

We have also used many other remedies in these parvules, and find them very convenient for grading the dose to any desired quantity; a saving of trouble in mixing and compounding, and what is very desirable, have found them in every instance to be pure, reliable and efficient in action. Their parvules of aloin are also excellent, as four of them can be taken with the same purgative effect as a like number of cathartic pills, and for administration to children, they are very convenient. Parvules of calomel, one-tenth grain, given in doses of two every two hours until five or six doses are taken, comprising one-half grain of calomel only, produce bilious evacuations and move the liver in a manner more satisfactory than ten grains of calomel as ordinarily given. As a class of remedies, we regard these parvules as very convenient and satisfactory. W.

**Cinchonia Alkaloid.**—Messrs. Powers & Weightman, of Philadelphia, have done a good work for the profession, and for the country, by the manufacture and by the introduction of the various alkaloids of the bark—distributing gratuitously to physicians throughout the country pure samples of these preparations for test and experiment. We have from time to time been furnished with these samples, and have both tested them ourselves and handed them out to others for trial in practice. On more than one occasion we have reported results from the experiments thus made with these agents.

The cinchonidia has proven an efficient anti-periodic; so the sulphate of cinchonia, and not less so the *cinchonia alkaloid* more recently introduced. A very excellent combination is the cinchonia mixture containing a portion of sugar of milk and bicarb. soda. It is nearly tasteless, and admirably suited to children and to delicate stomachs. It is rendered soluble by the natural acids of the stomach, or its solubility may be assured by the use of acid drinks.

Our observations in the use of this agent fully confirm the reports of others as to its anti-periodic properties, which may be regarded as little if any less than those of the sulphate of quinine. One intelligent practitioner pronounces the cinchonia mixture, above referred to, as specially adapted to nervous and delicate subjects, having noticed in a number of instances a peculiar soothing, anodyne effect—the patient sweetly sleeping through and beyond the time of chill, and awaking refreshed and free from fever.

The mixture has been found also a useful remedy in menorrhagia, when followed by elixir vitriol as the acid drink. It moderates the flow, equalizes the circulation, and warms up the extremities. In three cases of menorrhagia we have used the following combination with prompt success:

℞. Cinchonia alkaloid.................gr. xxx.

Elix. vitriol.........................ʒ ij.

Fluid ext. ergot.....................℥ ss.

Aquæ cinnamoni....................℥ iij. M.

S. One teaspoonful every three hours.

**Diphtheria.**—Dr. D. H. Chase, in Medical Brief, says:

In the past three years I have treated nearly two hundred cases of diphtheria, as follows:

℞ Tr. chloride iron........................... 1 ounce.
Potas. chlorate............................. 1 drachm.

Mix. Sig. Give five to twenty drops in a swallow of water every four hours, and if throat is badly affected, use a swab of the pure tincture of iron and glycerine, once daily for three days.

For fever:

℞ Tr. aconite................................ 10 drops.
Tr. gelseminum........................... 20 drops.
Tr. phytolacca........................... 20 drops.
Water.................................... 4 ounces.

Mix. Sig. One teaspoonful every hour.

With this treatment I have not lost a case.

**Treatment of Burns.**—If the epidermis has not been removed, mild antiphlogistic means should be used, as laudanum and lead water. If it has been destroyed, soothing ointments are very comforting to the parts, as oxide of zinc ointment; anything, however, that will prevent the contact of the air with the excoriated surface, and soothe the exposed nerve filaments, can be used. —*Mar. Med. Journ.*

Nothing better than melted lard mixed with an equal quantity of the white of an egg, into a bland creamy substance. It is always at hand and gives prompt relief to pain.—Ed. *S. Med. Record.*

**Poisoning by Rhus Toxicodendron.**—Dr. J. B. Murfree, in Medical Brief, says:

I have noticed several remedies for poisoning by rhus, or the poison oak vine. Permit me to call attention to the black wash (U. S. Dis.)—calomel and lime water. I have used it for several years past with invariable success. I do not believe in specifics, but this remedy approaches as nearly being a specific as any I know of.

**Aconite.**—Dr. T. J. Fentress, in Medical Brief, says :

We find aconite in most all febrile and inflammatory affections truly an indispensable agent, particularly so in those cases characterized by an imperfect capillary circulation, feeble but frequent pulse, cool extremities, without complications, then this remedy has a fine effect alone.

But often complications exist or arise requiring other agents in combination to meet these indications. Let us take as an example a case of fever, with a strong, full and frequent pulse, and a marked tendency to the brain as shown by a flushed face, bright eyes and contracted pupils. We then give :

| | | |
|---|---|---|
| ℞ | Tinct. aconite rad.................... | 20 drops. |
| | Tinct. gelseminum (green root)....... | 60 drops. |
| | Aqua ................................ | 4 ounces. |

Mix. Sig. Dose—one teaspoonful every hour is truly specific to this condition.

Take the opposite condition : a fever with a quick, feeble pulse, imperfect capillary circulation, extremities cool, face pale, eyes expressionless, pupils dilated with a marked tendency to coma. We then give the following :

| | | |
|---|---|---|
| ℞ | Tinct. aconite......................... | 20 drops. |
| | Tinct. belladon ...................... | 30 drops. |
| | Aqua ................................ | 4 ounces. |

Mix. Sig. Dose—one teaspoonful every hour is truly specific to this condition.

In sthenic fever or inflammations, with a full, open, bounding pulse, active capillary circulation, give :

| | | |
|---|---|---|
| ℞ | Tinct. aconite......................... | 30 drops. |
| | Tinct. verat. (Norwood's)............ | 20 drops. |
| | Spts. nit. dulc......................... | 1 drachm. |
| | Aqua,.................................. | 4 ounces. |

Mix. Sig. Dose—one teaspoonful every hour will soon quiet the storm and place the patient in a favorable condition for subsequent treatment.

And this, in fact, is our leading treatment in all cases of pneumonia presenting the indications above referred to, and with a success too, that we never achieved under any other form of medication, and decidedly more pleasant and acceptable to the patient, which is quite an important item in the account.

**Oxalate of Cerium in Vomitiug of Pregnancy.**—Dr. Image, in Practitioner, says :

In severe cases I give oxalate of cerium every four hours for the first day, beginning the first dose half an hour before the patient rises, and then, as improvement takes place, diminishing it to three times a day, but always giving the first dose of the day before the patient moves from the horizontal position—a point to which I attach much importance. The formula I employ is :

| | | |
|---|---|---|
| ℞ | Cerii oxalatis.............................................. | grs.x. |
| | Pulv. trag. co ............................................ | grs.x. |
| | Tinct. aurantii .......................................... | ʒss. |
| | Aquam ad................................................. | ℥j. M. |

S. Shake and give one teaspoonful.

DR. J. C. BOOZER, of South Carolina, writes: "I was called to a little girl between one and two years old, who is subject to convulsions once a month, or, as her friends stated, with every change of the moon. Supposing I might relieve the attack by purging the patient, I administered calomel; but it had no perceptible effect. Bromide of potassium was also given repeatedly without relieving the case. A blister was then applied to the nape of the neck, and relief at once followed; and there has been no return of the convulsions. Can any one inform me why these attacks come on with the change of the moon?"

[Periodicity is a feature which pertains to many affections, particularly those of a nervous character. Epilepsy and epileptiform convulsions are not unfrequently marked by periodical returns at longer or shorter intervals. If the period should be four weeks, it must necessarily fall upon some one of the moon's phases, and from time immemorial the moon has been made responsible for many of the ills and wants of life. The word *Lunatic* (*Luna*, the moon), as applied to crazy people, originiated in this moon superstition. The idea has been long since exploded, and the word lunatic now applies, both in medicine and in law, to all forms of mania or mental derangement except idiocy.—ED.]

**Dog-fennel** (*Anthemis Cotula.*)—Dr. I. T. Suggs, of Texas, writes: "During the war an old lady told me that *dog-fennel* was a certain cure for chronic diarrhœa, and that there was no danger of giving too much of it. She told me of very bad cases she had cured with it. It was not long till I had a case which appeared almost hopeless. I gathered the tops and blooms of the weed (the weed was just matured), made a strong decoction, gave a teacupful about sundown; his bowels did not trouble him all night. Afterwards gave a tablespoonful three times a day. In forty-eight hours his evacuations were perfectly natural—he was cured. This is a sample of a great many cases. I was living near Tupelo, Mississippi, where soldiers were frequently camped from the evacuation of Corinth to the close of the war. We then used a great many domestic remedies, because we could get no others. I am satisfied that there might be a valuable extract made from dog-fennel. I have been using this remedy in my practice ever since the war; it is good for the diarrhœa of children; it makes a very pleasant syrup."

**Boracic Acid Ointment.**—This ointment is used in University College Hospital, as an application for burns, and is made according to a formula of Mr. Godlee, as follows:

| | |
|---|---|
| Boracic acid in fine powder | 1 part. |
| White wax | 1 part. |
| Paraffin | 2 parts. |
| Almond oil | 2 drops. |

Melt the wax, paraffin and oil, with a gentle heat; then add the acid, and continue stirring until it remains of uniform consistence. Before using, it should be reduced to a soft mass by rubbing it in a cold mortar.

**Trichinæ in the Flesh of Geese.**—Sixty soldiers of the garrison of Thionville lately fell sick of trichinosis, and two of them died. It has been ascertained that the disease arose, not from pork, but from the flesh of geese which they had eaten.

